# Family Income, Cumulative Risk Exposure, and White Matter Structure in Middle Childhood

**DOI:** 10.3389/fnhum.2017.00547

**Published:** 2017-11-13

**Authors:** Alexander J. Dufford, Pilyoung Kim

**Affiliations:** Department of Psychology, University of Denver, Denver, CO, United States

**Keywords:** family income, white matter, diffusion tensor imaging (DTI), cumulative risk, middle childhood

## Abstract

Family income is associated with gray matter morphometry in children, but little is known about the relationship between family income and white matter structure. In this paper, using Tract-Based Spatial Statistics, a whole brain, voxel-wise approach, we examined the relationship between family income (assessed by income-to-needs ratio) and white matter organization in middle childhood (*N* = 27, *M* = 8.66 years). Results from a non-parametric, voxel-wise, multiple regression (threshold-free cluster enhancement, *p* < 0.05 FWE corrected) indicated that lower family income was associated with lower white matter organization [assessed by fractional anisotropy (FA)] for several clusters in white matter tracts involved in cognitive and emotional functions including fronto-limbic circuitry (uncinate fasciculus and cingulum bundle), association fibers (inferior longitudinal fasciculus, superior longitudinal fasciculus), and corticospinal tracts. Further, we examined the possibility that cumulative risk (CR) exposure might function as one of the potential pathways by which family income influences neural outcomes. Using multiple regressions, we found lower FA in portions of these tracts, including those found in the left cingulum bundle and left superior longitudinal fasciculus, was significantly related to greater exposure to CR (β = -0.47, *p* < 0.05 and β = -0.45, *p* < 0.05).

## Introduction

Socioeconomic disadvantage has adverse effects on multiple aspects of the cognitive and psychological development of children. Lower childhood family income has been associated with more behavioral problems (Brooks-Gunn and Duncan, 1997), difficulties with language and cognitive development (Noble et al., 2007; Hackman and Farah, 2009), and higher rates of psychopathology (Bradley and Corwyn, 2002; Gilman et al., 2002). The search for plausible biological mechanisms underlying the relationship between socioeconomic disadvantage and child developmental outcomes has begun to examine brain development (Hertzman, 1999). Using magnetic resonance imaging (MRI), it is possible to examine the relationship between socioeconomic disadvantage, and macrostructural and microstructural variations in the brain. The neuroimaging literature investigating the relationship between socioeconomic disadvantage and brain structure has primarily focused on gray matter (Hanson et al., 2011, 2015b; Luby et al., 2013; Noble et al., 2015). However, less is known about the relationship between socioeconomic disadvantage and white matter structure in the brain. Thus, the current study investigated the relationship between family income and white matter organization in children.

While gray matter is primarily involved in the computational processes in the brain within regions, white matter is involved in the signaling among brain regions (Matute and Ransom, 2012). White matter has been demonstrated to play a role in a wide variety of processes including memory, language, and emotion regulation (Fields, 2008). Maturation of white matter in childhood has been associated with increases in cognitive functioning (Nagy et al., 2004; Mabbott et al., 2006), language development (Wong et al., 2011; Urger et al., 2015), and emotion regulation (Versace et al., 2015). Greater white matter fractional anisotropy (FA) across several white matter tracts in children is associated with more efficient processing in several domains including working memory (Vestergaard et al., 2011), executive functioning (Ursache and Noble, 2016), and language processing (Rosselli et al., 2014; Celesia and Hickok, 2015). Children living in families experiencing low income exhibit lower performance in these domains (Hoff, 2003; Hackman et al., 2010; Raver et al., 2016). Therefore, differences in white matter organization among brain regions involved in these processes are a potential mechanism for income-related variations in neurocognitive and affective development.

Recently, researchers have started to examine the relationship between socioeconomic disadvantage and white matter organization as measured by FA. FA values measure white matter organization by quantifying the degree of anisotropic diffusion (a value from 0 to 1, with 1 reflecting fully anisotropic diffusion) along an axon (Beaulieu, 2009). Using a voxel-wise approach across the whole brain in adults, greater socioeconomic disadvantage was associated with lower FA in portions of several tracts involved in cognitive and affective functioning. These areas included the uncinate fasciculus, superior longitudinal fasciculus, pontine crossing tract, corona radiata, cerebellar and cerebral peduncles, stria terminalis, and sagittal stratum (Gianaros et al., 2012). Across a wide age range (3–21), using a tract-based approach, lower FA was observed in the right parahippocampal cingulum and right superior corticostriate tract of children and adolescents experiencing lower socioeconomic status (Ursache and Noble, 2016). In the current study, we focused on middle childhood as a developmental time period in which there are significant developmental changes occurring for white matter structure (Paus et al., 1999; Schmithorst et al., 2002). Middle childhood is typical when symptoms of internalizing disorders (e.g., depression and anxiety) that are more prevalent in children of families experiencing low income begin to manifest (Feng et al., 2008; Shanahan et al., 2014). Additionally, these symptoms may predate clinical diagnosis (Costello et al., 2005; Thapar et al., 2012). Therefore, it is critical to investigate the relationship between family income and white matter organization in this period to examine potential neural risk markers for children experiencing socioeconomic disadvantage before any symptoms of internalizing symptoms may arise or worsen.

Furthermore, little is known about potential underlying processes that could account for the expected adverse consequences of childhood socioeconomic disadvantage and white matter organization. Children growing up in families experiencing low income are more likely to experience physical stressors such as noise, crowding, and poorer housing quality as well as psychosocial risk factors such as family turmoil, violence, and family separation (Brooks-Gunn and Duncan, 1997; Evans, 2004; Brody et al., 2012; Raver et al., 2015). Children living in families experiencing low income are far more likely to experience multiple of these stressors at one time. The cumulative risk (CR) model has been conceptualized to measure multiple risk factors as well as to capture the potential additive effects of risks on child development (Felner et al., 1995; Greenberg et al., 1999; Burchinal et al., 2000; Evans, 2003; Herrenkohl et al., 2003; Evans and Kim, 2010; Evans et al., 2013). CR has been proposed and tested as a potential mechanism that captures the extent to which multiple physical and psychosocial risk factors may overwhelm the developmental processes of a child and impact cognitive, social, and affective development. CR has been consistently found to mediate the associations between family income and child outcomes (Sameroff, 2006; Obradovic et al., 2012; Pressman et al., 2012; Evans et al., 2013), including relationships with allostatic load in children and adolescents (Evans, 2003; Evans et al., 2007), behavioral problems (Appleyard et al., 2005; Trentacosta et al., 2008), and emotion dysregulation (Kliewer et al., 2017). However, less is known about CR as a pathway through which socioeconomic disadvantage is associated with children’s brain outcomes. Therefore, we conceptualized CR as a possible pathway through which socioeconomic disadvantage is associated with white matter organization.

More recently, studies suggest that CR exposure may be associated with brain structure and function. In one study, greater childhood CR exposure was associated with lower gray matter volume and functional reactivity in the amygdala for adults (Evans et al., 2015). Greater cumulative stress exposure has also been associated with smaller amygdala and hippocampus volumes in childhood (Hanson et al., 2015b). As the current study had a cross-sectional design, the use of a mediation model would introduce bias (Maxwell and Cole, 2007). Therefore, we explored the associations among socioeconomic disadvantage, CR, and white matter organization by examining whether portions of white matter tracts that are associated with family income may further be associated with CR.

The current study investigated the associations between socioeconomic disadvantage [as measured by income-to-needs ratio (INR)] and white matter organization in middle childhood as well as the role of CR exposure in white matter organization. We adopted a whole brain, voxel-wise approach in which the relationship between family income and white matter organization was assessed at each voxel in white matter tracts across the whole brain. Based upon the previous studies, we expect an association between INR and FA in a portion of the fronto-limbic tracts including the uncinate fasciculus and cingulum bundle. However, as network models and studies of connectivity become increasingly critical to understand brain function, it is important to examine the association between experiencing low family income and white matter organization across the whole brain. Therefore, we adopted a whole brain approach instead of a tract-based approach which is limited to a few *a priori* white matter tracts of interest. Furthermore, we predict that children experiencing low family income are also likely to be exposed to higher levels of CR exposure, and CR may further be associated with the white matter organization in the hypothesized tracts. We also conducted an exploratory analysis of the direct effect of CR in a whole brain model. This was to explore whether the effects of CR exposure would overlap with the effects of INR. As a second exploratory analysis, we examined the association between maternal education and white matter organization. Maternal education has been used as another proxy for socioeconomic position in studies of socioeconomic disadvantage and the brain (Jednoróg et al., 2012; Noble et al., 2012, 2015; Brito and Noble, 2014).

## Materials and Methods

### Participants

A total of 30 healthy children participated in this study. However, 3 participants were removed from the analysis due to high levels of artifact in their diffusion data (see section Diffusion Image Quality Control) reducing the sample size to 27 (see **Table 1** demographic information). Children were 8–10 years old and only one child per household was permitted to participate. Inclusion criteria included (1) INR below 6 (INR is calculated by dividing total family income by the poverty threshold that is specified by the United States Census Bureau, an INR of 6 represents upper middle income), (2) living with their biological mother for at least 50% of their time, and (3) fluency in English. Participants were excluded from the study if they (1) had a past or present diagnosis of a neurological disorder, (2) were currently being treated for a psychiatric disorder, (3) had ferrous metal or other MRI contraindications that would prevent scanning, (4) had an IQ score below 70 (assessed by the Wechsler Abbreviated Scale of Intelligence, WASI) (Wechsler, 1999), and (5) had past or current treatment with psychoactive medication.

**Table 1:** Income-to-needs ratio, maternal education, and demographic information for the sample.

	*N*	Mean ± *SD*	Range
Income-to-needs ratio		2.16 ± 1.40	0.00–4.95
Maternal education (years)		15.14 ± 2.59	9–20
Child age (years)		8.66 ± 0.67	8–10
Child sex (female)	16		
Child race/ethnicity			
White/Caucasian	15		
Black/African-American	7		
Hispanic	2		
Multi-racial	3		


### Procedure

We recruited participants and their biological mothers by contacting research volunteers from a database at the University of Denver and we placed flyers advertising the study in the Denver metro area public schools and anti-poverty programs. Socioeconomic diversity of the sample was insured by initially screening 149 families for eligibility for the study, and targeting low- and middle-income families. We intentionally oversampled low-income families, as this demographic was the main interest of the study. We screened 149 families, 47 of which participated in the home visit portion of the study. Of the families that participated in the home visit portion of the study, 30 participated in the MRI scan. Families that were interested in participating in the study contacted the laboratory via phone call. After participants were considered eligible, a visit was completed in which researchers visited the home of the participant. At the home visit, we conducted an interview with each mother as well as the WASI with each child. After completing the home visit, participants were invited for an MRI visit in which they completed a scan to obtain the diffusion-weighted data. If the interval between the home visit and MRI visit was more than 3 months, the family income was updated at the MRI visit. This study was carried out in accordance with the recommendations of the Institutional Review Board of the University of Denver. Written informed consent was obtained from the mother of each participant as well as written informed and verbal assent was obtained from each participant in accordance with the Declaration of Helsinki. The study protocol was approved by the Institutional Review Board of the University of Denver.

### Measures

#### Family Income

We used INR to assess family income; INR measures were calculated by dividing the total family income by the poverty threshold adjusted for the number of individuals living in the household as specified by the United States Census Bureau. INR was based upon on the parent-reported family income for the last 12 months. For the sample, 48% of the participants were considered low income (as defined by an INR ≤ 2).

#### CR Exposure

We calculated CR based upon previous studies by using the same measures and methods of data collection (Evans and Kim, 2007, 2012; Evans et al., 2007, 2013; Kim et al., 2013). CR exposure was measured using six environmental risk factors including three physical (crowding, noise, and housing quality) and three psychosocial factors (family turmoil, violence, and child separation from family). Crowding was measured by dividing the number of individuals living in the household (a resident is defined as living in the house at least three nights of the week) by the number of rooms in the house including bathrooms (this only included rooms frequented by the residents and did not include garages, attics, etc., unless it was regularly occupied such as in the case of a finished basement). Noise was assessed using a decibel meter (Leq) placed in the primary social space in the house (usually the living room) in which noise was monitored for a 2-h session during the home visit. Housing quality was assessed by a trained researcher using an observer-rated standardized scale (Evans et al., 2000) which collects data on structural defects, maintenance, childhood resources, safety hazards, and cleanliness.

For psychosocial risks, maternal responses were collected using the Life Events and Circumstances Checklist (Work et al., 1990; Wyman et al., 1991) to assess family turmoil, violence, and child separation from family. The checklist is comprised of 32 items which is administered in interview format by a trained research assistant. The interview includes multiple subscale items in which the respondent answers dichotomous (yes/no) questions for each of the three subscales (family turmoil, child separation from family, and violence). Within the Life Events and Circumstances Checklist, eight items ask about the child’s exposure to turmoil such as “your child was upset by family arguments.” Eight items asked about the child’s exposure to separation from the family such as “your child had to live with a relative or friend for a while.” Lastly, five items asked about the child’s exposure to violence such as “your child saw someone get badly hurt.” Based upon the responses of each item (1 points for yes as the response, 0 points for no as the response), scores were summed for each of the psychosocial domains.

For each of the six domains, risk was defined dichotomously (as 0 or 1) based upon a statistical criterion for the sample. Per domain, participants scored 1 indicating the presence of the risk if the score was in the top quartile (Wells et al., 2010; Evans and Kim, 2012; Evans and Cassells, 2014) across the whole sample. The scores across all the domains were summed to indicate each participant’s total CR score with values ranging from 0 to 6 (6 indicating the presence of all of the risk domains). The advantages to using the current approach to measuring CR has been reviewed in detail (Evans et al., 2013).

### MRI Acquisition

Diffusion-weighted data was acquired using a 3T Siemens TIM Trio MR scanner and a 32-channel head coil with the following parameters: 72 transversal slices were collected (anterior to posterior) each at 2 mm thick, TR = 9100 ms, TE = 86 ms, FOV = 256 mm, and voxel size = 2.0 mm × 2.0 mm × 2.0 mm. The diffusion-weighted images (DWIs) were acquired along 71 non-collinear directions, with a *b* factor of 1000 s/mm^2^, and one image with *b* = 0 s/mm^2^ as a reference volume.

### Diffusion Image Quality Control

Acquisitions of DWIs are particularly susceptible to multiple artifacts which can lead to biasing of the estimation of diffusion parameters especially when imaging pediatric samples. Visual quality control (QC) was conducted by a trained analyst; for this procedure, each volume of participant’s DWI acquisition was assessed for artifacts. The visual QC focused on signal dropout and image striping (‘Venetian blind’ artifact). For each participant, the number of volumes that were identified as containing artifact was recorded. Based upon the number of volumes that contained artifact per participant, participants’ DWI acquisitions were labeled as “poor” (more than 14 volumes or 20% contained artifact), “good” (1–14 volumes contained artifact), or “excellent” (no volumes contained artifact) (Roalf et al., 2016). Participants with DWI acquisitions in the “poor” category were excluded from the sample (3 participants); “good” and “excellent” acquisition (27 participants) were entered into the analysis. For the participants that were entered into the analysis, the mean number of volumes with artifact was 3.92 ± 3.08. However, we chose not to manually exclude the volumes with artifact as it has been shown to negatively impact the precision and accuracy when estimating FA (Chen et al., 2015).

### DTI Image Processing

Using diffusion imaging, the structural organization of white matter fibers can be measured *in vivo* and a tensor model can be fit to each voxel to estimate the fibers degree of FA. FA measures the directional dependence of water molecule diffusion and reflects the microstructural properties of organization including fiber density, myelination, and axonal caliber (Beaulieu, 2009). FA is measured from 0 to 1 in which greater values indicate greater fiber organization. Therefore, while some studies conceptualize FA as white matter integrity, we refer to it as a measure of white matter organization because FA is also sensitive to factors not reflecting fiber integrity such as crossing fibers, partial voluming, etc. (Alexander et al., 2007). FA is the most widely used DTI index (Assaf and Pasternak, 2008) and has the largest body of research examining its associations with cognitive and affective functioning (Nagy et al., 2004; Sexton et al., 2009). Therefore, we chose to focus our analysis on FA as a measure of white matter organization. Our analysis followed the standard Tract-Based Spatial Statistics (TBSS) pipeline (Smith et al., 2006). Diffusion image processing was conducted using the Oxford Center for Functional MRI of the Brain’s (FMRIB) Software (FSL version 5.0)^1^. DWI acquisitions were corrected for motion and eddy current distortion. Motion parameters were extracted during this process and no images had translation that exceeded 3 mm or rotation exceeding 5° for more than 0–5% of the DWI acquisition (Liu et al., 2010). Skull stripping was conducted for the DWI using FSL’s Brain Extraction Tool (BET). While several studies have used BET for brain extraction in pediatric populations (Nordahl et al., 2011; Group, 2012; Nie et al., 2013), it is considerably less accurate (Vân Phan et al., 2017). Therefore, we conducted visual QC on the brain extracted images; for any images that failed the visual inspection such as erosion of brain tissues or inclusion of non-brain tissues, the image was reprocessed using the ‘robust’ brain center estimation option in BET for recursive calls of bet2 to improve brain extraction (Fagiolo et al., 2008; Yang et al., 2014). After visual QC and reprocessing, all brain extractions were deemed acceptable for inclusion in the analysis. Using FSL’s *DTIFit* function, diffusion tensor models were fit at each voxel using the default standard linear regression option which captures the magnitude and single orientation of diffusion; FA, mean diffusivity, 1st eigenvector, 2nd eigenvector, 3rd eigenvector, 1st eigenvalue, 2nd eigenvalue, and 3rd eigenvalue images were generated for each participant. FA images were realigned into FMRIB standard-space FA image, and were registered into MNI152 standard space. We chose to use a standard template as opposed to a study-specific template for TBSS-derived measures of white matter organization as it has been demonstrated to have increased reliability for the standard template (Madhyastha et al., 2014) especially when sample sizes are limited. While there is no clear age cutoff for using an adult versus a pediatric template, several studies have used the standard adult template provided with FSL for pediatric studies with similar age ranges (some even younger than 8 years old) than our sample (Barnea-Goraly et al., 2009; Odegard et al., 2009; Ou et al., 2014; Blanken et al., 2017; Gunbey et al., 2017). The mean FA image was thinned to create a skeleton to represent white matter tracts that were common to all the participants in the sample and was thresholded at 0.2 to exclude FA values from gray matter or cerebrospinal fluid. The threshold value of 0.2 has been used in several studies using pediatric samples (Billeci et al., 2012; Carr and Pike, 2012; Liu et al., 2013; Saygin et al., 2013).

### Diffusion Image Analysis

A whole brain, voxel-wise analysis of the FA skeleton was conducted using TBSS to examine the relationship between INR and FA. We used FSL’s *Randomize* to conduct a non-parametric permutation test on the imaging data using the Threshold-Free Cluster Enhancement (TFCE) option to identify regions of white matter in which FA was associated with INR. TFCE is generally more conservative compared to cluster-based thresholding, and avoids the need for an arbitrary initial cluster-forming threshold (Smith and Nichols, 2009). *Randomize* was run using 5000 permutations and the TFCE *p*-value images (which were corrected for multiple comparisons across space using family-wise error correction) were examined for significant clusters using a threshold of *p* < 0.05. Clusters identified as significant were localized anatomically using the Johns Hopkins University (JHU) White Matter Tractography Atlas (Mori et al., 2008).

Multiple linear regression analysis was used to examine the associations between INR and the FA values extracted from the whole brain analysis to investigate whether the relations observed in the whole brain model were due to the possible confounds of age, sex, ethnicity, or whole brain FA. These covariates were included in the *post hoc* analyses rather than in the whole brain model to avoid potential issues of multicollinearity and statistical overcontrol in the whole brain model. INR was correlated with age and was trending toward significance for ethnicity (see section Demographic Variables). Although INR was not significantly correlated with the sex of the participants, white matter structure has been demonstrated to show significant sex differences in middle childhood (Barnea-Goraly et al., 2005; Simmonds et al., 2014). We included whole brain FA as a covariate to insure that the results were not driven by global FA and were due to localized FA values (Tromp et al., 2012; Lant et al., 2016).

To assess the relationship between the significant clusters and CR scores, FA values from each cluster were extracted using FSL *Cluster* tool and entered into SPSS for further analysis. We used a multiple regression approach in which we examined the association between CR and the FA extracted from each cluster in a separate regression. In each regression, we included age, sex, ethnicity, and whole brain FA as covariates.

For the exploratory analyses, we also conducted a whole brain, voxel-wise analysis using *Randomize* and TFCE. For the whole brain CR model, CR exposure was used as the predictor and for the whole brain maternal education model, maternal education (in years) was used as the predictor. Because these were exploratory analyses, we tested the results at *p* < 0.05 FWE corrected, and then explored the results at a more lenient threshold, *p* < 0.05 uncorrected.

## Results

### Demographic Variables

We examined the relationship between INR and demographic variables for the sample. A statistically significant difference was found for INR between ethnicities (ethnicity was defined as White/Caucasian, Black/African-American, Hispanic, and Multi-racial) as determined by a one-way ANOVA, *F*(3,23) = 3.13, *p* < 0.05. While the overall ANOVA was significant, a Tukey’s *post hoc* test for the ANOVA revealed that INR was trending toward significance for the White/Caucasian group and the Black/African-American group (2.6 ± 1.27, *p* = 0.59). As INR was trending toward significance for the pairwise comparison between these two groups, we decided to include ethnicity as a covariate which was binary encoded (as White and Non-white) for all the *post hoc* regression analyses. For the sample, INR was significantly correlated with age such that older participants tended to have higher INR (*r* = 0.48, *p* < 0.05). While whole brain FA was not associated with INR, it was included as a covariate to insure global FA was not driving the association between INR and FA. Additionally, sex, ethnicity, and age were included as covariates in the *post hoc* analysis models. Although INR was not significantly correlated with the sex of the participants, white matter structure has been demonstrated to show significant sex differences in middle childhood (Barnea-Goraly et al., 2005; Simmonds et al., 2014). Thus, child sex was included as a covariate in the *post hoc* analyses. INR was not correlated with maternal education for the sample. For the description of the demographics of the sample, see **Table 1**.

**Table 2** depicts the mean, SD, range, and top quartile cutoff for each domain of CR. While our sample was significantly smaller, the CR scores were within similar ranges of studies with much larger sample sizes in which the top quartile was used as the statistical criterion for determining CR (Evans et al., 2007, 2015). Participants with scores below this value were assigned a 0 indicating the absence of the risk. There was no significant association between demographic variables and CR exposure. A multiple regression was used to assess the association between INR and CR exposure; controlling for the sex, age, and ethnicity of the participants, the association was significant (β = -0.70, *p* < 0.01, *f*^2^ = 0.64). Without covariates, the correlation between INR and CR remained significant (*r* = -0.53, *p* < 0.01).

**Table 2:** Descriptive information of the cumulative risk factors.

Risk factor	Mean	*SD*	Range	Quartile
CR (total)	1.7	1.46	0.00–5.00	NA
Crowding	0.44	0.20	0.00–1.00	0.54
Noise (Leq)	54.67	6.14	31.8–71.40	57.2
Housing quality	0.48	0.29	0.00–1.08	0.73
Family turmoil	2.66	1.83	0.00–6.00	3.00
Violence	1.03	1.01	0.00–3.00	1.00
Family separation	2.11	1.01	0.00–5.00	2.00


*Quartile values represent the top quartile (statistical criterion) for the sample.*

### INR and White Matter Organization

**FIGURE 1:**
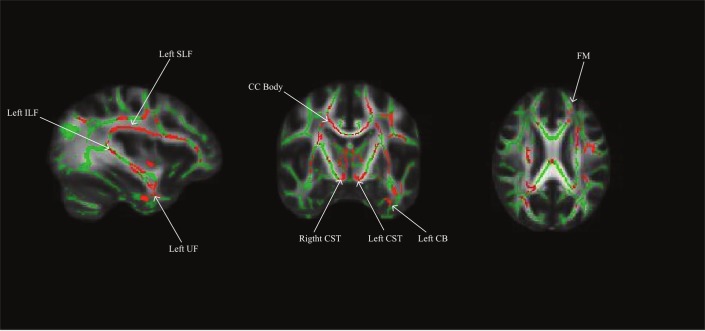
Whole brain model results for income-to-needs ratio (INR) as a predictor of FA. Green regions indicate the FA template for the sample, and red regions indicate a significant association at *p* < 0.05 FWE corrected (UF, uncinate fasciculus; CB, cingulum bundle; SLF, superior longitudinal fasciculus; ILF, inferior longitudinal fasciculus; CC, corpus callosum; CST, corticospinal tract; FM, forceps minor).

Using a whole brain, voxel-wise approach, we tested the relationship between INR and FA across the group FA image. There was a positive relationship between INR and FA such that lower INR was associated with lower FA in a portion of the left uncinate fasciculus, left cingulum bundle (hippocampus), bilateral superior longitudinal fasciculus, bilateral corticospinal tracts, left inferior longitudinal fasciculus, left anterior thalamic radiation, the forceps minor, fornix, and the body of the corpus callosum (see **Figure 1** and **Table 3** for the description of the clusters), *p* < 0.05 FWE corrected.

**Table 3:** Cluster information from the TBSS analysis for income-to-needs ratio analysis.

Cluster	Voxels	*X*	*Y*	*Z*	JHU Label
1	938	109	161	96	Forceps minor
2	771	132	127	92	L Superior Longitudinal Fasciculus
3	759	91	102	47	R Corticospinal Tract
4	660	68	109	113	R Superior Corona Radiata
5	556	83	102	60	R Corticospinal Tract
6	483	123	107	62	Fornix
7	457	112	108	124	L Corticospinal Tract
8	420	124	117	37	L Cingulum (hippocampus)
9	417	135	108	64	L Inferior Longitudinal Fasciculus
10	181	133	154	80	L Uncinate Fasciculus
11	129	100	113	101	Body of Corpus Callosum
12	33	94	115	78	Anterior Thalamic Radiation


*Clusters are identified from the analysis of income-to-needs ratio. Clusters are significant at *p* < 0.05 FWE corrected. MNI coordinates reflect the peak voxel for each cluster. Clusters were anatomically defined using the Johns Hopkins University (JHU) White Matter Tractography Atlas as available in FSL.*

### INR, CR, and White Matter Organization

**FIGURE 2:**
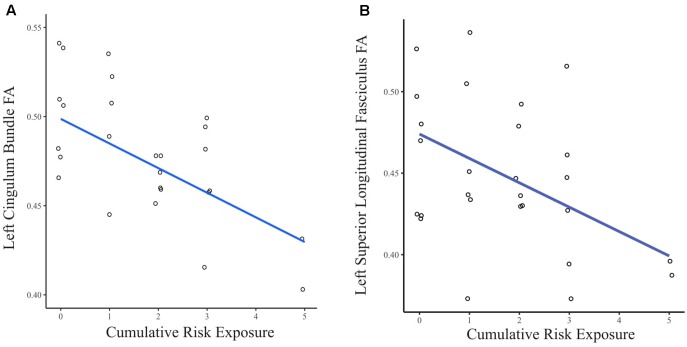
**(A)** Scatter plot of the relationship between CR exposure and FA in the left cingulum bundle. **(B)** Scatter plot of the relationship between CR exposure and FA in the left superior longitudinal fasciculus. Data points are jittered to allow visualization of overlapping values.

When the FA values were extracted from the whole brain INR model, greater CR exposure was associated with lower FA in clusters in several white matter tracts (see **Figure 2**). Using separate multiple regressions with age, sex, ethnicity, and whole brain FA as nuisance covariates, we identified a significant relationship between FA values and CR exposure in clusters of the left cingulum bundle (β = -0.47, *p* < 0.05, *f*^2^ = 0.69), left superior longitudinal fasciculus (β = -0.45, *p* < 0.05, *f*^2^ = 0.76), body of the corpus callosum (β = -0.40, *p* < 0.05, *f*^2^ = 1.04), left corticospinal tract (β = -0.41, *p* < 0.05, *f*^2^ = 0.82), and right corticospinal tract (pontine) (β = -0.44, *p* < 0.05, *f*^2^ = 1.20). There was no significant relationship found between CR exposure and the left uncinate fasciculus, left inferior longitudinal fasciculus, a portion of the left superior longitudinal fasciculus, a portion of the right corticospinal tract, forceps minor, anterior thalamic radiation, and fornix.

For the exploratory analysis of CR and white matter organization, no significant results were observed at the threshold of *p* < 0.05 FWE corrected. However, for the exploratory analysis (at *p* < 0.05, uncorrected), we observed significant clusters in portions of white matter tracts similar to the analysis of INR such as the left cingulum bundle and left superior longitudinal fasciculus.

### Maternal Education and White Matter Organization

Using a similar whole brain model as used in the INR model, we tested the association between maternal education and white matter organization. For the exploratory analysis of maternal education (with *p* < 0.05 FWE corrected), we did not observe any significant results. We observed similar results as the INR analysis with clusters in portions of the left uncinate fasciculus, left cingulum bundle, and left superior longitudinal fasciculus at *p* < 0.05 uncorrected.

## Discussion

We examined the relationship between family income and white matter organization in middle childhood by using a whole brain, voxel-wise approach. This approach allows examination of the relationship between family income and FA at each voxel of white matter in the whole brain. With this analytic approach, we found that for several regions of white matter including those located in the left uncinate fasciculus, left cingulum bundle, and left superior longitudinal fasciculus, that lower family income was associated with lower structural organization assessed by FA. The clusters of white matter that were associated with family income are part of white matter tracts that have been identified in tract-based studies to be involved in a range of cognitive, socio-emotional, and motor processes (Schermuly et al., 2010; Vestergaard et al., 2011; Von Der Heide et al., 2013). Additionally, we examined the relations between significant regions of white matter tracts associated with family income and exposure to CR. Lower family income was associated with increased levels of CR exposure. For FA from several significant clusters including those in the left cingulum bundle and left superior longitudinal fasciculus, exposure to more CR factors was associated with lower FA.

There was a large overlap between the clusters identified to be associated with socioeconomic status in a study of adults (Gianaros et al., 2012), especially in the cingulum bundle and uncinate fasciculus, and clusters associated with INR in the current study in middle childhood. Our findings suggest that the association between family income and regional white matter organization evolves earlier in life, and is present at least by middle childhood. This is important because variations in white matter organization may be a potential neural risk marker for psychopathology that may develop in adolescence and adulthood. While lower FA among lower income children occurred across several regions in white matter tracts in the brain, it is critical to understand the possible functions of the individual tracts they were found in, the regions they connect, and their potential relationship with socioeconomic disadvantages in developmental functioning.

We found a significant cluster in the uncinate fasciculus. The uncinate fasciculus is a bi-directional white matter tract that connects regions of the limbic system (including the amygdala), orbitofrontal cortex, and Brodmann’s Area 10 with the anterior temporal lobes (Von Der Heide et al., 2013). Lower FA in the uncinate fasciculus has been implicated in several mental disorders that are more prevalent in children growing up in low-income families, such as generalized anxiety (Tromp et al., 2012) and depression (Zhang et al., 2012). Lower family income was also related to lower FA in a portion of the left cingulum bundle, similar to findings of a previous study in children from ages 3–21 using a tract-based approach (Ursache and Noble, 2016). This white matter tract connects the cingulate gyrus to the dorsal hippocampus (Azmitia and Segal, 1978), and also has projections to the dorsolateral and dorsomedial prefrontal cortex (Croxson et al., 2005). Lower white matter organization in the cingulum bundle has also been observed in severe childhood adversity. Children exposed to maltreatment showed lower right cingulum bundle FA, which was then associated with the development of major depression and substance abuse in adolescence (Huang et al., 2012). Additionally, lower FA in the left cingulum bundle in previous research has been implicated in parental abuse and subsequent depression in young adults (Choi et al., 2009). Thus, further studies are also needed to examine the relationship between family income, uncinate fasciculus and cingulum bundle FA, and risks for internalizing disorders in childhood.

In the current study, lower INR was also related to lower FA in cluster in the fornix. The fornix is a C-shaped bundle of fibers which is a major output of the hippocampus and has been implicated in alterations in recall memory functioning (Gaffan, 1974). Our finding may provide convergent evidence with the association between family income and lower gray matter volume in the hippocampus in childhood (Hanson et al., 2011; Luby et al., 2013). Lower family income was also associated with lower FA in a portion of the superior longitudinal fasciculus. The superior longitudinal fasciculus projects from the frontal lobe to the parietotemporal association areas (Makris et al., 2005). The superior longitudinal fasciculus is implicated in a wide variety of functions including working memory (Vestergaard et al., 2011) and language (Mandonnet et al., 2007). Lower FA in the superior longitudinal fasciculus may contribute to difficulties in the areas of language processing and working memory which are among the most sensitive outcomes associated with childhood disadvantage (Bradley and Corwyn, 2002; Hackman and Farah, 2009). Other significant clusters from the whole brain analysis included: the forceps minor, corticospinal tracts, superior corona radiata, inferior longitudinal fasciculus, and body of the corpus callosum. While the association between the functions of these tracts and socioeconomic disadvantage remains unclear, future studies including functional MRI and behavioral measures may help elucidate this relationship.

In the current study, we did not observe the associations between maternal education and white matter organization, unless the statistical threshold was significantly relaxed as in the exploratory analysis. Moreover, previous studies of socioeconomic disadvantage and brain structure that have examined both the association of family income and maternal education within the same sample have found different results for each analysis (Hanson et al., 2011; Luby et al., 2013; Noble et al., 2015). Some suggest that family income and maternal education may be measuring unique mechanisms, and/or risk factors such as family income may be more sensitive to individual differences in material resources (Bradshaw and Main, 2016), while maternal education may be more sensitive to individual differences in factors such as parenting style (Carr and Pike, 2012). Future studies, with a larger sample size, will be needed to examine the common and unique associations between INR, maternal education, and white matter structure in childhood.

We were also interested in exploring the feasibility of CR exposure as one explanation for the expected adverse relation between childhood socioeconomic disadvantage and white matter organization. As mentioned earlier, the data from this study is cross-sectional, so it is inadvisable to conduct a formal mediation analyses (Maxwell and Cole, 2007; Maxwell et al., 2011). However, there is value in showing whether or not CR exposure is associated with both income and white matter organization, two prerequisites for mediation to operate. Lower family income was associated with greater CR exposure, which measures exposure to multiple stressors including crowding, noise levels in the home, housing quality, family turmoil, family separation, and violence. Several studies, both with children and adults, have consistently shown a mediating role of CR in the associations between socioeconomic disadvantage and negative developmental outcomes (Evans and English, 2002; Evans and Kim, 2010, 2012; Wells et al., 2010; Atkinson et al., 2015).

This study extended the literature by finding, among the regions of white matter tracts associated with lower family income, that CR was further linked to lower organization in clusters identified among regions of several white matter tracts. This provides evidence for a potential mediating role of CR for the white matter development among children experiencing socioeconomic disadvantage. Thus, future studies using a prospective design would be critical to examine changes in family income, white matter (scans at multiple time-points), and CR and then to relate them to developmental outcomes such as psychopathology and cognitive functioning. Further, for CR to operate as a mediator of the association between socioeconomic disadvantage and white matter organization, it will be critical to examine possible underlying biological mechanisms. Several biological mechanisms have been proposed to be associated with both socioeconomic disadvantage and developmental outcomes such as allostatic load, hypothalamic-pituitary-adrenal axis activity (including cortisol), and markers of inflammation (Evans, 2003; Evans and Kim, 2007; Danese et al., 2009). However, future studies will need to examine the associations between CR, biological pathways, white matter organization, and developmental outcomes.

Our findings are not without limitations, and should be interpreted as an initial investigation of childhood disadvantage, white matter organization, and the possible role of CR exposure in this relationship. First, socioeconomic disadvantage was calculated based upon the last 12 months of the participant’s life. Therefore, we cannot conclude whether the results observed are due to the current socioeconomic status of each participant or the impact of low family income since birth. Future studies need to examine the role of the chronicity of poverty exposure and its relationship to the development of white matter organization. Second, our sample size was relatively small compared to other studies examining the association between socioeconomic disadvantage and white matter (Ursache and Noble, 2016). Studies with larger sample sizes will be needed to replicate these findings. Third, this study focused on the chronic stress associated with low family income, however, we acknowledge that several other factors likely play a role in the relationship between income and brain structure. As discussed, the potential neurobiological mechanisms of the relationship between family income and white matter organization remain elusive. Further studies, including contributions from the animal literature, will be needed to examine potential mechanisms contributing to the lower white matter organization that was observed. Possible mechanisms include higher levels of glucocorticoids (van der Werff et al., 2014; Cox et al., 2015), exposure to toxins (Gray et al., 2013), nutritional deprivation (Kant and Graubard, 2012), as well as lack of cognitive stimulation (Conger et al., 2010; Lipina et al., 2013). Lastly, a correlation between INR and CR may raise a concern regarding the potential issues of ‘double dipping’ (Kriegeskorte et al., 2009). However, our main interest of the analysis is not the correlations *per se* but whether the CR has a potential to be a pathway by which family income is associated with the white matter organization. As mentioned, it would be inadvisable to conduct a mediation analysis (Maxwell and Cole, 2007) for our study due to it being cross-sectional. However, by restricting our analysis to the clusters associated with FA, and examining the association with CR, we provide evidence that among those regions with a significant association with INR, greater levels of CR are associated with lower FA. The FA in portions of some of the tracts was also associated with CR exposure in an exploratory whole brain analysis.

In summary, we provide evidence of a relationship between family income and white matter organization in clusters located in regions across several white matter tracts in the brain. These tracts are involved in a multitude of cognitive and affective processes, including those neurocognitive domains in which children experiencing socioeconomic disadvantage have shown difficulties compared to their middle-income counterparts. These tracts play roles in both cognition and affective regulation, and have been implicated in several psychiatric disorders more prevalent in children experiencing low income. As such, these children may show similar white matter organization prior to developing symptoms or symptoms worsening in adolescence. Future directions for this research will provide insight into multiple aspects of the association between socioeconomic disadvantage and white matter organization. First, as white matter development, especially in fronto-limbic circuitry, continues until early adulthood (Uda et al., 2015), we can follow up with this cohort and repeat neuroimaging in adolescence and early adulthood and can examine associations between INR and white matter development, as well as examine whether CR mediates this relationship. Second, one study found child maltreatment was associated with lower organization in the uncinate fasciculus which was further linked to later internalizing symptoms (Hanson et al., 2015a). While examining the relationship between white matter organization and symptoms of psychopathology was beyond the scope of this study, future studies will attempt to examine this relationship as well as the prospective relationship between white matter organization and specific measures of cognitive functioning such as executive functioning, working memory, and measures of affective functioning such as emotion regulation. Third, while we focused on FA as our measure of white matter organization for this study, future studies are needed to examine the association between INR and other DTI-based measures including mean diffusivity, radial diffusivity, and axial diffusivity to provide a comprehensive analysis of INR and white matter indices.

The findings related to family income may also inform preventative and intervention programs by more efficiently targeting specific neurocognitive domains by understanding properties of the underlying neural networks that are involved. Additionally, CR exposure was inversely related to structural organization across several white matter tracts involved in multiple neurocognitive processes. This finding highlights the potential impact of multiple, co-occurring stressors being related to less FA in several clusters of white matter across several networks. Therefore, preventative and intervention programs targeting the multiple stressors associated with socioeconomic disadvantage may have widespread impact on the connectivity of distributed networks in the brain.

## Author Contributions

PK and AD designed the study. AD analyzed the data. AD and PK wrote the manuscript.

## Conflict of Interest Statement

The authors declare that the research was conducted in the absence of any commercial or financial relationships that could be construed as a potential conflict of interest.

## Footnotes

1 http://www.fmrib.ox.ac.uk/fsl
